# Multiple-Organ Complement Deposition on Vascular Endothelium in COVID-19 Patients

**DOI:** 10.3390/biomedicines9081003

**Published:** 2021-08-12

**Authors:** Paolo Macor, Paolo Durigutto, Alessandro Mangogna, Rossana Bussani, Luca De Maso, Stefano D’Errico, Martina Zanon, Nicola Pozzi, Pier Luigi Meroni, Francesco Tedesco

**Affiliations:** 1 Department of Life Sciences, University of Trieste, 34127 Trieste, Italy; ldemaso@units.it; 2 Laboratory of Immuno-Rheumatology, Istituto Auxologico Italiano, IRCCS, 20095 Milan, Italy; pdurigutto@units.it (P.D.); pierluigi.meroni@unimi.it (P.L.M.); 3 Institute for Maternal and Child Health, IRCCS Burlo Garofolo, 34137 Trieste, Italy; alessandro.mangogna@burlo.trieste.it; 4 Department of Medical, Surgical and Health Sciences, University of Trieste, 34149 Trieste, Italy; bussani@units.it (R.B.); sderrico@units.it (S.D.); martina.zanon@virgilio.it (M.Z.); 5 Edward A. Doisy Department of Biochemistry and Molecular Biology, Saint Louis University School of Medicine, St. Louis, MO 63104, USA; nicola.pozzi@health.slu.edu

**Keywords:** COVID-19, complement activation, multi-organ deposition, classical pathway, spike protein

## Abstract

Increased levels of circulating complement activation products have been reported in COVID-19 patients, but only limited information is available on complement involvement at the tissue level. The mechanisms and pathways of local complement activation remain unclear. The aim of this study was to investigate the deposition of complement components in the lungs, kidneys, and liver in patients with COVID-19 patients and to determine the pathway/s of complement activation. We performed immunofluorescence analyses of autopsy specimens of lungs, kidney, and liver from 12 COVID-19 patients who died of acute respiratory failure. Snap-frozen samples embedded in OCT were stained with antibodies against complement components and activation products, IgG, and spike protein of SARS-CoV-2. Lung deposits of C1q, C4, C3, and C5b-9 were localized in the capillaries of the interalveolar septa and on alveolar cells. IgG displayed a similar even distribution, suggesting classical pathway activation. The spike protein is a potential target of IgG, but its uneven distribution suggests that other viral and tissue molecules may be targeted by IgG. FB deposits were also seen in COVID-19 lungs and are consistent with activation of the alternative pathway, whereas MBL and MASP-2 were hardly detectable. Analysis of kidney and liver specimens mirrored findings observed in the lung. Complement deposits were seen on tubules and vessels of the kidney with only mild C5b-9 staining in glomeruli, and on the hepatic artery and portal vein of the liver. Complement deposits in different organs of deceased COVID-19 patients caused by activation of the classical and alternative pathways support the multi-organ nature of the disease and the contribution of the complement system to inflammation and tissue damage.

## 1. Introduction

The vast majority of individuals infected by the new coronavirus SARS-CoV-2 manifests mild to moderate disease and usually recover within a few weeks. However, some of them, for unknown reasons, experience a severe form of disease and require intensive care treatment [1,2]. The respiratory tract is considered the main target of SARS-CoV-2 that infects epithelial cells in the trachea and bronchi and pneumocytes in the lungs, causing pneumonia, that in more severe cases, progresses to acute respiratory distress syndrome [3]. Nonetheless, other organs may also be involved, including the heart, kidneys, and liver [4], due to the wide distribution of the virus receptor ACE-2 [5,6]. Consistent with the multi-organ nature of this complex disease, analysis of a large number of COVID-19 patients has revealed that while two thirds of severe cases manifest acute respiratory distress syndrome, one third develop heart and kidney failure, as well as liver dysfunction [7].

Hyperinflammation is a common feature in symptomatic COVID-19 infection and is characterized by infiltration of inflammatory cells in the lungs and other infected organs, particularly evident in severe forms of the disease that may lead to the appearance of autoinflammatory and autoimmune phenomena [8]. This process is the result of dysregulated response of the innate immune system [9] and is sustained by pro-inflammatory cytokines released by macrophages and other cells at tissue sites [10]. However, the analysis of severe cases has shown that the clinical severity of the disease is not always associated with increased levels of pro-inflammatory cytokines and other markers of inflammation, such as C-reactive protein [11].

Complement (C) has emerged as a potential key contributor to the development of inflammation and tissue damage in COVID-19 patients, with the release of the pro-inflammatory peptides C3a and C5a that help to recruit leukocytes to the lung and other infected tissues and the assembly of the terminal complex that damage vascular endothelium and promotes thrombus formation [12,13]. We have reported increased levels of C5a and sC5b-9 related to the severity of disease and not always associated with a parallel increase in acute phase proteins in COVID-19 patients group [14,15]. Similar findings have been reported by Gao et al. in the preprint server medRxiv and Valenti et al. [16,17]. Carvelli and colleagues have recently suggested the involvement of C5a-C5aR1 axis in the pathogenesis of SARS-CoV-2 infection and the potential benefit of the therapeutic blockade of this interaction [18]. Elevated C activation products have been found significantly elevated in patients with respiratory failure [19] and complement hyperactivation has been reported to be associated with chromosome gene 3 cluster variation and non-O blood group patients with severe COVID-19 [17]. More direct evidence for the contribution of C to tissue damage was obtained from postmortem analysis of two lung and three skin biopsy specimens of COVID-19 patients that revealed deposits of C activation products C4d, C3d, and C5b-9 in the lung inter-alveolar septal microvessels and in the skin vasculature [20]. The finding of MASP2 localized in the pulmonary inter-alveolar septa of one COVID-19 patient led Magro and coworkers to conclude that C is activated through the lectin pathway [20].

The aim of the present investigation is to analyze the distribution of early and late C components in the lung, kidney, and liver of COVID-19 patients with the intent to clarify the pathway/s of C activation and to document the involvement of tissues other than lung as targets of C attack.

## 2. Materials and Methods

### 2.1. Study Group

The study group comprised 12 patients, 7 females and 5 males aged 72 to 97, referred to the University Hospital in Trieste (Italy). Two patients were admitted to the Intensive Care Unit where they received intubation and mechanical ventilation. The remaining patients were followed in other medical sections of the hospital, including Infectious Diseases and Geriatrics wards, or in nursing homes from which they were transferred to one of the hospital wards when the clinical conditions deteriorated. The diagnosis of SARS-CoV-2 infection suspected on the basis of the clinical symptoms were confirmed by RT-PCR analysis of nasopharyngeal swab. This work was approved by the Ethical Committee of the Regione Friuli Venezia Giulia, Italy (Prot. N. 0025523/P/GEN/ARCS). Patients of the control group were selected as they were of similar age range and co-morbidities to the COVID-19 patients.

### 2.2. Tissue Sample Collection

Limited autopsies from 12 SARS-CoV-2-positive and 3 SARS-CoV-2-negative cases with a similar age range were performed by an experienced pathologist, and samples were collected from the lungs, kidneys, and liver for this study. Three or more tissue blocks were obtained from selected areas of the three organs and fixed for 24 h in 10% buffered formalin. Parts of these samples were paraffin-embedded, and 3-µm thick sections were stained with hematoxylin and eosin for histological examination. Detailed analysis of tissue abnormalities in 41 autopsies, including the 9 autopsies reported in this work, has been previously published [21]. Briefly, histologic analysis of the lungs of all COVID-19 patients revealed vascular thrombosis, extensive damage of the tissue structure, with marked edema and intra-alveolar fibrin deposition. Conversely, both the kidney and liver presented near-normal structure, with only some age-related alterations.

### 2.3. Immunofluorescence Analysis

Formalin-fixed tissue samples were snap-frozen and embedded in OCT medium (Diagnostic Division; Miles Inc., Elkhart, IN, USA). Tissue sections of 7 µm were stained with the following primary antibodies (5 μg/mL): goat anti-human IgG (Sigma-Aldrich, Milan, Italy), goat anti-C1q and C4 (The Binding Site, Birmingham, UK), goat anti-C3 (Quidel, San Diego, CA, USA), and anti-Factor B (FB) (Cytotech, Sandwich, UK); rabbit anti-MBL (Sigma-Aldrich, Milan, Italy), anti-SARS-CoV-2 Spike S2 (Sino Biological, Wayne, PA, USA) and anti-human von Willebrand Factor (vWF) (Dako, Bolzano Italy); murine monoclonal antibody against C9 neo-antigen (aE11), kindly provided by prof. T.E. Mollnes, (Oslo, Norway). The following FITC-conjugated secondary antibodies were used to reveal bound antibodies: rabbit anti-goat IgG (Sigma-Aldrich, Milan, Italy), goat anti-rabbit IgG and anti-mouse IgG (Dako), CF405M-labeled goat anti-rabbit IgG (Sigma-Aldrich, Milan, Italy) was used to reveal the anti-vWF IgG. The slides were mounted with the Mowiol-based antifading medium (Sigma-Aldrich, Milan, Italy), and the Images were acquired with the fluorescence microscope Leica DM2000 equipped with DFC420 camera (Leica, Milan, Italy) [22]. At least three random sections of tissue samples collected from each case were examined independently by three observers. Cases with moderate to strong staining intensity were considered as positive, while those with weak staining intensity were defined as weakly positive, and all the others with undetectable staining were scored as negative.

### 2.4. Western Blot Analysis

The presence of specific proteins in tissue samples was confirmed by western blot performed as previously described [23]. Unfixed tissue samples from Patient 12 and control C were snap-frozen and embedded in OCT medium (Diagnostic Division; Miles Inc., Elkhart, IN, USA). Ten tissue sections (10 µm) were lysed in RIPA buffer, sonicated and dialyzed against PBS, and the protein concentration was measured using Bradford Reagent (Sigma-Aldrich, Milan, Italy). Protein samples (20 µg) were separated by SDS-PAGE and analyzed by western blot using antibodies employed for the immunofluorescence analysis. Alkaline phosphatase-labeled secondary antibodies and BCIP-NBT were used to reveal the bound antibodies. Membranes were analyzed by ChemiDoc MP Imaging System (Biorad, Segrate, Italy) and the intensity of the stained bands was quantified using ImageJ (Fiji-NIH).

### 2.5. ELISA for C5b-9 Analysis

The level of C5b-9 complex was measured in the protein samples used for the western blot analysis according to a previously described method with slight modification [24]. Briefly, the wells of ELISA plates (Costar Costar, Milan, Italy) were coated with a monoclonal antibody to C5b-9 neoantigen (A239, Quidel, San Diego, CA, USA), and incubated with the protein samples (20 µg/mL). The bound C5b-9 was revealed using biotin-labeled anti-C5 antibody (Quidel), alkaline phosphatase-labeled streptavidine (Sigma), and PNPP (Sigma). Data were expressed as OD 405 nm.

## 3. Results

### 3.1. Clinical Data

The main clinical findings in COVID-19 and control patients are summarized in Table 1 and Table 2.

They all manifested symptoms related to pneumonia, which was confirmed by High Resolution Computer Tomography analysis in 11 of them. Radiological assessment could not be performed in one patient due to the very old age. Cancer and diabetes were the most frequent co-morbidities, and eight also experienced heart disease, while six had neurological problems. Hypoxemia evaluated by blood gas analysis was particularly severe in all patients, with PaO2/FiO2 ratio of 100 or less. Increased levels of the inflammatory and coagulation markers CRP, ferritin, and D-dimer and lymphopenia were commonly observed at diagnosis (Supplementary Table S1) and were more marked before death (Table 2). Unfortunately, the presence of antibodies to SARS-CoV-2 and systemic complement activation were not investigated. As per hospital guidelines, the patients were treated with low molecular weight heparin and steroids to prevent clot formation and to control hyperinflammation. Death occurred after 22 ± 11 days of hospitalization (mean ± standard deviation) because of acute respiratory failure.

### 3.2. C Deposition in the Lungs

**Figure 1:**
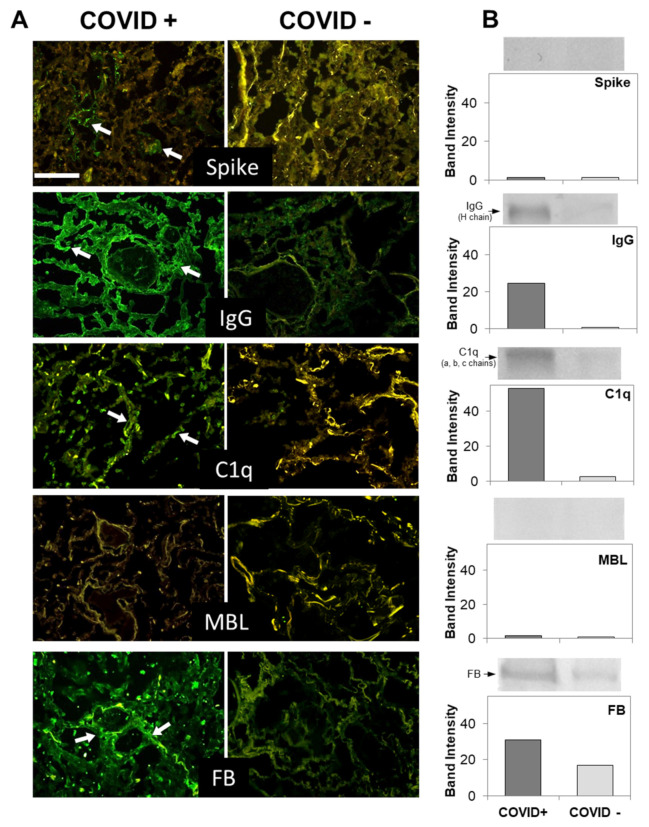
(**A**) Detection of spike and complement components in lung sections by immunofluorescence analysis. The panel shows representative immunofluorescence images obtained from the analysis of several sections of lung autopsy samples from 12 SARS-CoV-2-positive and 3 negative cases. The sections were stained to reveal the presence of spike protein, IgG, MBL, C1q, and Factor B (FB) (highlighted by a white arrow), as explained in materials and methods, and examined by three independent observers. Scale bar = 50 µm. (**B**) Evaluation of spike and complement components by western blot in the lungs of a COVID+ and a COVID− patients. The columns represent the staining intensity of the western blot bands quantified using ImageJ (Fuji-NIH). Western blot bands of spike protein, IgG, C1q, MBL and FB are shown on top of the corresponding columns.

Data from previous immunohistochemical studies have suggested the involvement of the lectin pathway in C activation in COVID-19 patients [16,20]. To confirm these findings, we stained lung tissues with antibodies against MBL and, to our great surprise, we failed to detect tissue deposits of this protein, except for occasional faint staining of MBL in vascular thrombi (Figure 1).

To determine if C was activated by other initiators of the lectin pathway, such as ficolins and collectins, we examined the autopsy specimens for the presence of MASP-2, which is strictly required for the activation of the lectin pathway; but, as for MBL, the staining was negative (data not shown). We next investigated the activation of the classical pathway. Extensive deposition of C1q was found in the capillaries of the interalveolar septa and, to a lesser extent, on alveolar cells (Figure 1).

Double staining of lung tissue section with antibodies to vWF and C1q revealed co-localization of the two proteins on vascular endothelium and also expression of C1q on cells that did not stain for vWF (Figure S1).

**Figure 2:**
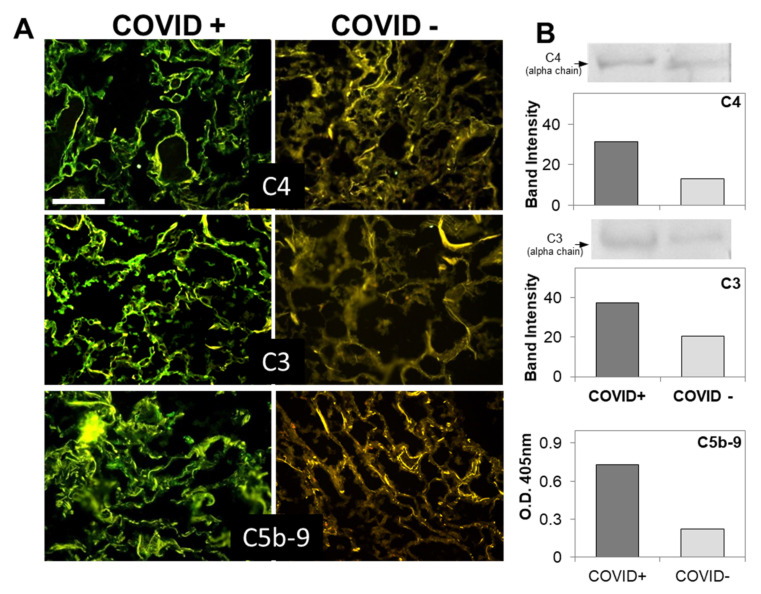
(**A**) Detection of complement components in lung sections by immunofluorescence analysis. The panel shows representative immunofluorescence images obtained from the analysis of several sections of lung autopsy samples from 12 SARS-CoV-2-positive and 3 negative cases. The sections were stained to reveal the presence of C4, C3, and C5b-9, as explained in materials and methods, and examined by three independent observers. Scale bar = 50 µm. (**B**) Evaluation of complement components by western blot in the lungs of a COVID+ and a COVID− patients. The columns represent the staining intensity of the western blot bands quantified using ImageJ (Fuji-NIH). Western blot bands of C4 and C3 are shown on top of the corresponding columns. C5b-9 levels were measured by ELISA and expressed as OD readings at 405 nm.

Since the distribution of C1q deposition was similar than that of IgG, C4, and C3 in all cases (Figure 1 and Figure 2 and Table 3), we concluded that the classical pathway is a common route of C activation in the lungs of our COVID-19 patients. We also looked into the activation of the alternative pathway by staining the sections for FB, which was easily detectable (Figure 1), and concluded that, in addition to the classical pathway, the alternative pathway must be playing an important role in C activation in these patients. In agreement with previous data by Magro et al. and Gao et al. [16,20], we found that the terminal complex C5b-9 localizes along the alveolar wall and on the interalveolar microvascular vessels (Figure 2). The spike protein was detected on alveolar cells and interalveolar septal capillaries in 5 out of 12 specimens, but, unlike IgG and C components, displayed an uneven distribution (Figure 1 and Figure 2). Analysis of control autopsy specimens showed background staining for C components and activation products, IgG and spike protein (Figure 1 and Figure 2). To further clarify the degree of protein deposition on lung tissue, western blot analysis and ELISA were used for quantitative evaluation of individual C components, IgG, and spike protein in the proteins extracted from the tissue. As shown in Figure 1B and Figure 2B, a marked difference was observed in the amount of protein deposited on COVID-19 positive and control tissue, while both the spike protein and MBL were hardly detectable.

To summarize, activation of both classical and alternative pathways documented by deposition of C1q, C4, C3, and FB was observed in nine cases, while activation of either pathway was found in two cases. C components and C activation products were undetectable in a single case, except for a mild staining for FB and IgG (Table 3).

### 3.3. C Deposition in Kidney and Liver

**Figure 3:**
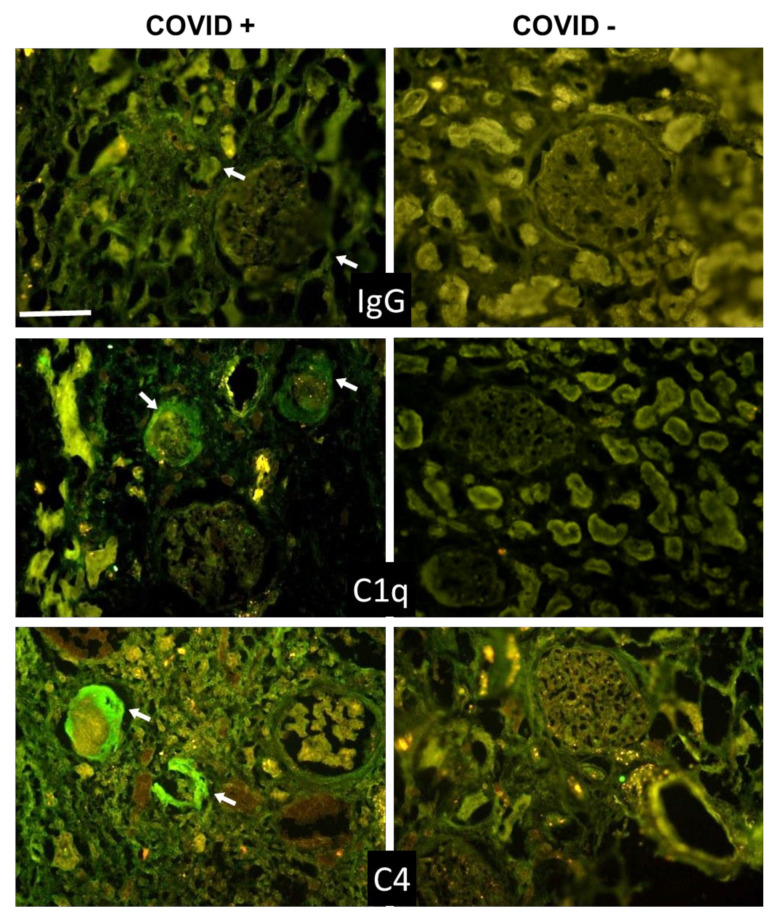
Detection of IgG, C1q, and C4 in kidney sections by immunofluorescence analysis. The panel shows representative immunofluorescence images obtained from the analysis of several sections of kidney autopsy samples from 12 SARS-CoV-2-positive and 3 negative cases. See legend to Figure 1 for further details. White arrows show IgG, C1q, and C4 periglomerular deposition. Scale bar = 50 µm.

**Figure 4:**
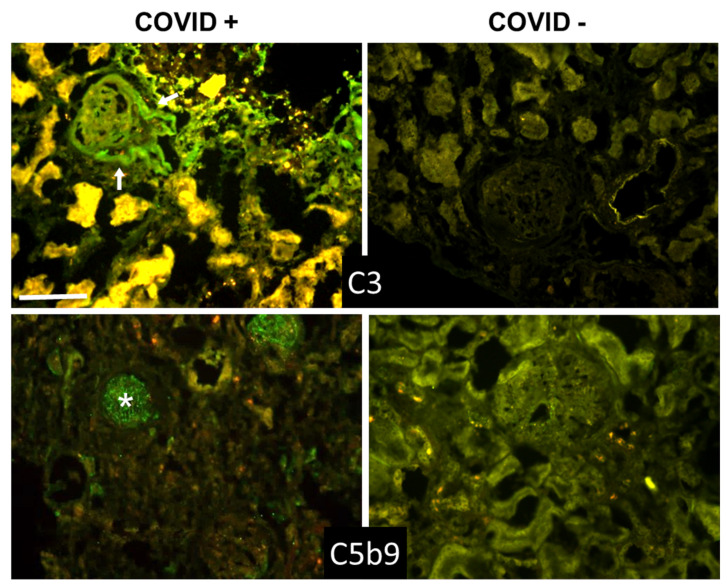
Detection of C3 and C5b-9 in kidney sections by immunofluorescence analysis. The panel shows representative immunofluorescence images obtained from the analysis of several sections of kidney autopsy samples from 12 SARS-CoV-2-positive and 3 negative cases. See legend to Figure 1 for further details. White arrows in C3 image show periglomerular deposition; * in C5b-9 image shows glomerular staining. Scale bar = 50 µm.

**Figure 5:**
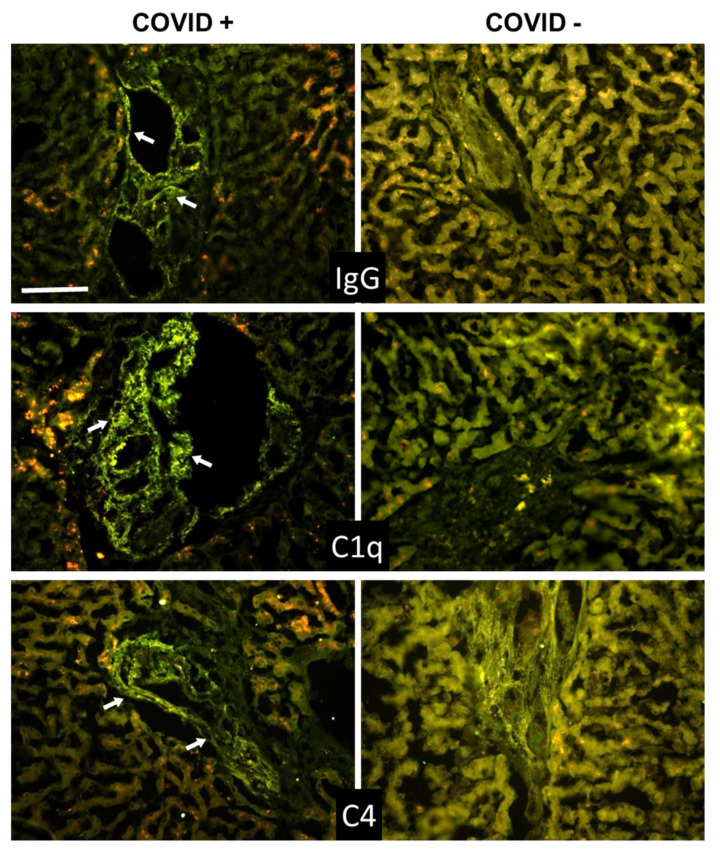
Detection of IgG, C1q, and C4 in liver sections by immunofluorescence analysis. The panel shows representative immunofluorescence images obtained from the analysis of several sections of liver autopsy samples from 12 SARS-CoV-2-positive and 3 SARS-CoV-2-negative cases. See legend to Figure 1 for further details. White arrows indicate vessels in the portal area. Scale bar = 50 µm.

**Figure 6:**
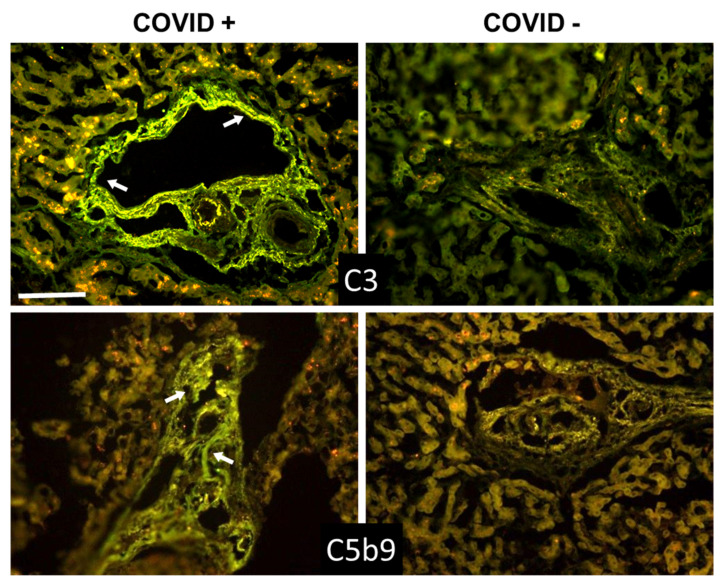
Detection of C3 and C5b-9 in liver sections by immunofluorescence analysis. The panel shows representative immunofluorescence images obtained from the analysis of several sections of liver autopsy samples from 12 SARS-CoV-2-positive and 3 SARS-CoV-2-negative cases. See legend to Figure 1 for further details. White arrows indicate vessels in the portal area. Scale bar = 50 µm.

Beyond the lungs, signs of C activation were searched for in the kidney and liver, two organs that may be affected by the virus. The data summarized in Table 4 and Table 5 show that C and IgG deposition patterns observed in kidneys (Figure 3 and Figure 4) and liver (Figure 5 and Figure 6) were remarkably similar to those seen in the lung, suggesting that C activation may follow similar pathways in all these organs. The detection of C5b-9, both in the kidney and liver, supports the progression of C activation to completion in the majority of cases. Interestingly, IgG and C deposits in both kidney and liver were observed in the absence of overt pathological lesions.

A detailed analysis of kidney sections revealed the presence of IgG and C components on tubules, vessels, and in the periglomerular areas, and mild C5b-9 staining in glomeruli (Figure 3 and Figure 4). Staining for the spike protein was found in 5 out of 12 cases and was seen in the tubules and some small vessels, but undetectable in the glomeruli.

As for the liver, the portal area represents the main site of C localization, which was observed primarily on the hepatic artery and portal vein (Figure 5 and Figure 6), supporting the vascular involvement in C-mediated tissue damage in COVID-19 patients. The spike protein was found to localize in the portal area of 5 out of 12 cases.

## 4. Discussion

C activation products detected in the plasma of COVID-19 patients have recently emerged as useful markers to monitor the progression of the disease from early to advanced stages. However, it is important to acknowledge that these methods fail to report on the extent of local C activation, whose information is key to a better understanding of the mechanisms underlying tissue damage and disease progression. This work investigates C deposition in 12 COVID-19 patients at tissue level. Our results show that C deposits are present in the lungs, kidneys, and liver, providing additional evidence for multi-organ injuries in the disease [5,7,25], and suggesting that C-mediated damage can potentially affect other organs besides the lungs.

C deposits were found in the lungs of the majority of our patients, localized on the wall of the interalveolar septal capillaries and, to some extent, also on alveolar epithelial cells. Accumulation of C deposits in these anatomical structures is likely to cause lung alterations, including alveolar damage, interstitial edema, and vascular injury responsible for the impaired gas exchange [21,26]. It is also likely to exacerbate the inflammatory response associated with tissue damage. The intensity of C staining was generally related to the extent of pulmonary pathological alterations with particular reference to the severity and diffusion of the inflammatory response. There was one notable exception in a patient who had massive inflammation with loss of lung architecture and yet no sign of C deposition. The reason for this observation is not apparent, but it may well be that in this case, the inflammatory process is induced and sustained by pro-inflammatory cytokines known to be involved in the pulmonary inflammation in COVID-19 patients [27].

Considering that all our patients were elderly people aged more than 70, it is not surprising that they had multiple comorbidities that may have contributed to induce C activation together with SARS-CoV-2 infection. However, while this possibility cannot be excluded, it should be pointed out that the comorbidities in the majority of cases did not specifically affect the respiratory tract, and when they did in three cases, C deposits were either reduced or not much different compared to all the other cases. This finding is consistent with the observation that C and IgG deposition is negligible or undetectable in the lung of COVID-19 negative patients, despite the clinical and radiologic signs of pathologic conditions such as COPD and pulmonary embolism documented in two patients. A point of concern was that the post-mortem period varying between 1 and 3 days prior to autopsies might have had an impact on the degree of tissue C deposition, but the autopsies of COVID-19 and control groups, with the only exception of case 6, were performed within the same range of post-mortem interval. An interesting finding of this study was that the C deposits were not restricted to the lungs but were also detected in kidneys and liver of the same patients, in line with the accepted view that COVID-19 is a multi-organ disease [4,5,25]. Both these organs shared with the lungs a common feature of predominant, though not exclusive, C localization on blood vessels, suggesting the contribution of locally deposited C activation products to microvascular injury and thrombosis.

Published reports have suggested that C is mainly activated through the lectin pathway in patients infected by SARS-CoV-2 [28,29]. The viral spike protein has been proposed as a possible candidate to trigger the lectin pathway, given the high degree of glycosylation that may enable this protein to interact with the recognition molecules of the pathway, though the evidence to support this possibility is lacking. Yu and colleagues [30] have recently published data showing that the spike protein activates the alternative pathway that can be prevented by inhibition of factor D and C5. The other viral protein that may be implicated in the activation of the lectin pathway is the N protein of SARS-CoV-2. Gao and colleagues [16] reported in a non-peer reviewed publication, data indicating that N protein binds MASP2 and enhances the cleavage of C4 and C3 induced by the immobilized MBL-MASP2 complex. They also showed that components of the lectin pathway were present in the pulmonary tissue of COVID-19 patients, although the protein deposits were hardly visible in the published figure, raising some doubt on the in vivo relevance of these results. Staining for MASP-2 was also observed by Magro et al. [20] in the post-mortem pulmonary analysis in COVID-19 patients, but the finding was limited to one out of five patients examined, and no information was given on the tissue localization of MBL and other recognition molecules of the lectin pathway. The faint or absent staining of our patients’ lung for MBL and MASP-2 cannot be attributed to the failure of the antibodies to recognize their targets, as both proteins were detected in the liver cells that synthesize these proteins, but rather indicates that the lectin pathway is not the main driver of C activation in our study group. However, we cannot exclude that the lectin pathway may be involved in the promotion of thrombosis, one the hallmarks of COVID-19 disease. Eriksson and coworkers [31] have recently reported increased levels of MBL in COVID-19 patients with thrombotic episodes that were correlated with the levels of plasma D-dimers, but not with the inflammatory process and organ alterations. These data are consistent with our recent observation that MBL interacts with beta2-glycoprotein expressed on endothelial cells and activates the lectin pathway, leading to MASPs-mediated cleavage of prothrombin and thrombin generation [32]. The pro-coagulant activity of MASPs may explain the beneficial effects observed in COVID-19 patients treated with Narsoplimab [33] which inhibits MASP-2, preventing C activation and thrombus formation, although the small number of treated patients and the concomitant use of other drugs represent important limitations recognized by the authors of this study.

The positive staining of the patients’ lung for C1q points to the classical pathway as an important route of C activation, likely triggered by the IgG that were widely distributed in the lungs of all patients examined. Unfortunately, the immunohistochemical analysis does not permit definition of the antigenic specificity of these IgG. However, it is tempting to speculate that the spike protein of SARS-CoV-2 present in several tissue samples is a potential target, although we cannot exclude that other viral and tissue antigens may also be recognized by the antibodies. The latter possibility was suggested by the reports of sequence homology between SARS-CoV-2 and human proteins-derived peptides published by different groups [34,35,36] and is supported in this study by the finding of spike protein-independent localization of IgG in the lung of COVID-19 patients. An important implication of these observations is that, because of cross-reactivity of human and viral proteins, the antibody response to SARS-CoV-2 may contribute to the pathogenesis of the disease, causing tissue damage possibly as a result of C activation. A similar conclusion was reached some years ago by Yang and colleagues [37] who were able to detect antibodies to primary human pulmonary endothelial cells and human pulmonary epithelial cell line in patients with SARS-associated coronavirus infection. More recently, Wang and colleagues [38] have reported a substantial increase of autoantibodies directed against immunomodulatory molecules responsible for dysregulation of immune functions in COVID-19 patients.

The detection of IgG and C1q in kidneys and liver is consistent with similar findings in the lung specimens and supports the conclusion that the classical pathway is the prevailing route of C activation, further amplified by the involvement of the alternative pathway, although the finding of MASP-2 deposits in 2 out of 9 COVID-19 renal biopsies by Pfister et al. [39] suggested the possible contribution of the lectin pathway. As hypothesized for C activation in the lung, the complex of antibody and spike protein documented in both the kidney [40,41] and liver [42] may be the initial trigger of C activation, followed by the intervention of additional factors that contribute to potentiate C activation with the progression of the disease severity.

## 5. Conclusions

In conclusion, this work has shown that antibodies and C deposits can be detected in post-mortem biopsy specimens of various organs, including the lung, kidney, and liver, collected from COVID-19 patients supporting the accepted view that this is multi-organ disease. In addition, evidence has been provided that C is preferentially activated through the classical pathway with the important contribution of the alternative pathway. Taken together, these data further support the involvement of C in COVID-19 pathogenesis and the use of therapeutic agents to prevent C-dependent tissue damage and sustained inflammatory response associated with this severe infectious disease.

## Figures and Tables

**Table 1:** Clinical findings.

Cases	Age	Sex	Respiratory Signs *	Comorbidities	Radiologic Findings	Time from Diagnosis to Death (Days)	Hospitalization (Days)	Postmortem (Days)
1	73	M	yes	Prostate cancer; Lung metastases; Pyonephrosis; Heart failure	Bilateral airspace opacites	24	23	1
2	82	M	yes	Diabetes; Bladder cancer; Hypercholesterolemia	Bilateral airspace opacites	49	18	2
3	80	M	yes	Encephalopathy; Urosepsis; Seizure	Bilateral airspace opacites	30	30	1
4	97	F	yes	Colon cancer	NA	3	14	1
5	90	F	yes	Hypertension; Diabetes	Pleural effusion left lung	48	48	1
6	77	F	yes	Dementia; Stroke; Melanoma	Bilateral airspace opacites	4	3	3
7	74	F	yes	Seizure; Paraplegia; Atrial fibrillation	Ground glass right lung; left lung lower lobe consolidation	ND	14	2
8	72	M	yes	BPCO; heart failure; Diabetes; stroke	Bilateral airspace opacites	27	27	2
9	79	F	yes	Heart failure; Bilateral fibrotorax	Emphysema; Airspace opacities right lung	27	27	2
10	80	F	yes	Relapsing urinary infection; Stroke with left hemiparesis; Heart failure; FA; Hypercholesterolemia	Pleural effusion	5	11	1
11	77	F	yes	Bilateral carotid stenosis; Hypothyroidism; Obesity; Bladder and right breast cancer; Chronic vascular encephalopathy	Bilateral airspace opacites	23	20	2
12	94	M	yes	BPCO; Asbestosis; Hypertension; FA; History of relapsing falls	Bilateral calcific fibrothorax; Ground glass right lung; Interstitial pneumonia	25	24	2
Control A	84	F	yes	Anemia; Obesity; Previous DVT with massive pulmonary embolism; Chronic cholestasis; Vascular encephalopathy with cognitive impairment	Pulmonary embolism; Pleural effusion; Right lung consolidation	ND	5	1
Control B	79	F	yes	Hypertension; Hypertensive-ischemic heart disease; Diabetes; Stroke; Esophagitis	Mild and widespread interstitial disease	ND	7	2
Control C	95	M	yes	COPD; Hypertension; FA; Cognitive impairment; MGUS	Right lung consolidation; Bilateral pleural effusion	ND	24	2

* Dyspnea, fever, cough; ND: Not Determined.

**Table 2:** Laboratory findings before death.

Cases	D-Dimer (µg/L)	CRP (mg/dL)	Ferritin (µg/L)	White Cells (n × 10^3^/µL)	Neutrophils (n × 10^3^/µL)	Lymphocytes (n × 10^3^/µL)	Platelets (n × 10^3^/µL)
1	17,180	15.6	2059	14.59	6.72	0.52	173
2	3020	30.6	2892	15.13	11.25	1.65	256
3	1330	30.2	90.9	12.98	9.59	2.45	2.84
4	1330	302.8	352	20.83	6.13	0.82	239
5	NA	9.1	85	12.68	11.21	0.43	461
6	6800	16.08	1608	5.57	3.89	0.76	231
7	2120	3.82	882	14.03	6.57	1.15	259
8	1800	210	755	14.19	13.51	0.31	83
9	430	15.7	1035	23.03	3.52	1.82	326
10	NA	4.97	244.6	7.69	5.74	2.19	355
11	1046	30.48	2146	17.79	19.91	1.26	222
12	1536	1.85	294.8	4.8	7.34	0.34	104
Controls	<500	<0.05	30–400	4.8–10.8	1.5–6.5	1.2–3.4	130–430

**Table 3:** Complement deposition in lungs.

	P1	P2	P3	P4	P5	P6	P7	P8	P9	P10	P11	P12								
**IgG**																				
**C1q**														P	**Postive**					
**MBL**																				
**Factor B**														W	**Weakly positive**					
**C4**																				
**C3**														N	**Negative**					
**C5b-9**																				
**General C activation**																				

**Table 4:** Complement deposition in kidneys.

	P1	P2	P3	P4	P5	P6	P7	P8	P9	P10	P11	P12								
**IgG**														P	**Postive**					
**C1q**																				
**C4**														W	**Weakly positive**					
**C3**																				
**C5b-9**														N	**Negative**					
**General C activation**																				

**Table 5:** Complement deposition in livers.

	P1	P2	P3	P4	P5	P6	P7	P8	P9	P10	P11	P12								
**IgG**														P	**Postive**					
**C1q**																				
**C4**														W	**Weakly positive**					
**C3**																				
**C5b-9**														N	**Negative**					
**General C activation**																				

## Data Availability

Not applicable.

## Supplementary Materials

The following are available online at https://www.mdpi.com/article/10.3390/biomedicines9081003/s1, Figure S1: Detection of vWF and C1q in lung sections by immunofluorescence analysis. The panel shows representative immunofluorescence images obtained from the analysis of several sections of lung autopsy samples from 12 SARS-CoV-2-positive cases. The sections were double stained to reveal the co-localization of vWF and C1q, as explained in materials and methods, and examined by three independent observers. White arrow indicate co-localized staining of the two antibodies; white arrowheads show C1q staining without anti-vWF binding. Scale bar = 50 µm.; Table S1: Laboratory findings after diagnosis.
