# Supplementary material for: Multiple-Organ Complement Deposition on Vascular Endothelium in COVID-19 Patients
Source: Biomedicines. 2021 Aug 12;9(8):1003. doi: 10.3390/biomedicines9081003 (PMC8394811; doi:10.3390/biomedicines9081003)

Table S1. Laboratory findings after diagnosis

Cases	D-dimer (μg/L)	CRP (mg/dL)	Ferritin (μg/L)	White cells (n x 10^3/μL)	Neutrophils (n x 10^3/μL)	Lymphocytes (n x 10^3/μL)	Platelets (n x 10^3/μL)
1	1940	5.9	570	7.65	5.67	1.25	214
2	690	6.33	349.4	4.8	3.96	1.24	170
3	1930	8.93	85.2	10.22	9.14	1.55	138
4	1330	0.36	352	8.33	6.71	0.84	214
5	NA	2.34	85	5.43	4.21	0.73	211
6	5450	13.3	1608	5.57	3.89	0.76	231
7	2120	7.47	888.9	11.34	9.10	1.24	237
8	600	5.82	527.4	3.94	3.2	0.5	128
9	560	2.42	1035	6.51	3.52	1.82	338
10	NA	2.24	244.6	8.42	5.74	2.19	390
11	546	10.58	267.4	13.99	12.31	0.78	332
12	1400	3.18	294.8	5.73	3.74	0.92	189

NA: Not Available

Figure S1

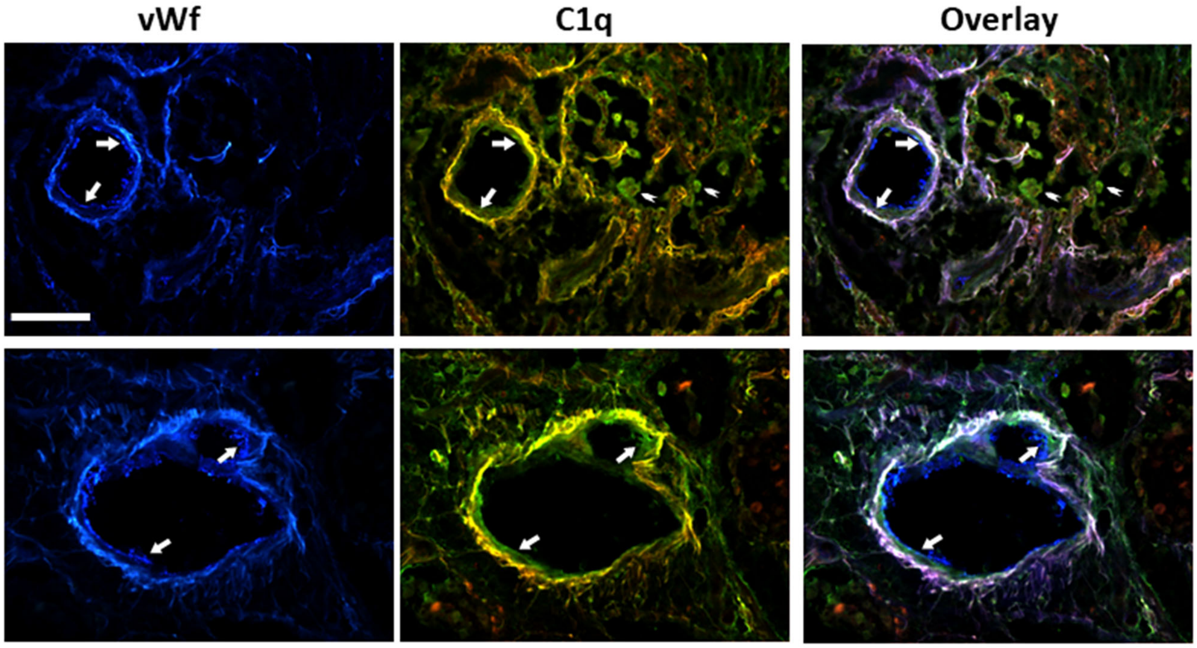

Supplement: Supplementary file 1 [file biomedicines-09-01003-s001.zip › biomedicines-1285684-supplementary.pdf]
